# Refractory Erythematous Rosacea: Combined Use of Paroxetine and Microdroplet Botulinum Toxin

**DOI:** 10.1111/jocd.71082

**Published:** 2026-07-21

**Authors:** M. A. Rocha, E. Bagatin

**Affiliations:** ^1^ Dermatologists From Federal University of São Paulo São Paulo Brazil

**Keywords:** botulinum toxins, erythematous rosácea, facial erythema, paroxetine, rosacea, type a

Dear Editor,

Wang et al., demonstrated that paroxetine 25 mg/day significantly improves persistent erythema and flushing in patients with rosacea refractory to conventional therapy, achieving Clinical Erythema Assessment (CEA) success in 42.9% versus 20.8% with placebo at week 12 [1]. These findings support the concept that selective serotonin reuptake inhibition may stabilize vasomotor dysregulation through serotonergic modulation, reducing sympathetic hyperreactivity and neurogenic inflammation [2]. In daily practice, however, monotherapy frequently provides incomplete control of rosacea. In a preliminary case series, we observed favorable and durable outcomes using a combined approach with oral paroxetine targeting central mechanisms and intradermal injection of onabotulinumtoxinA (BoNT‐A) acting peripherally on neurovascular and neurogenic pathways, suggesting a potential therapeutic synergy [3, 4].

The series included a 16‐year‐old girl with a two‐year history of persistent malar erythema without papules or pustules, negative autoimmune screening, and significant psychosocial distress. Previous treatments included gentle skincare, mineral sunscreen, topical oxymetazoline 1% with partial response and rebound erythema, oral carvedilol 6.25 mg twice daily without benefit, and six IPL sessions with limited improvement. Paroxetine 25 mg/day was initiated, leading to marked reduction in flushing and burning by 6 weeks and visible improvement in erythema at 12 weeks. To further stabilize vasomotor reactivity, intradermal BoNT‐A microdroplets, 1 U per point at 1‐cm intervals, with a total dose of 45 U, were administered over the erythematous area and repeated every 4 months during follow‐up. Near‐complete clearing was achieved by 4 months and sustained for 10 months. Adverse effects included mild transient bruising and 2 kg weight gain, which stabilized after tapering paroxetine to 10 mg/day [5]. No clinically relevant relapse was observed after paroxetine dose reduction during the reported follow‐up.

The series also included 28‐year‐old twin sisters who developed persistent centrofacial erythema and burning during the COVID‐19 pandemic. Both had failed multiple therapies, including doxycycline, oxymetazoline 1%, carvedilol, and repeated IPL and Nd:YAG 1064 nm sessions, with < 20% improvement; one patient discontinued doxycycline because of drug‐induced hepatitis. Severe heat intolerance markedly impaired daily activities. A combined protocol of paroxetine 25 mg/day and intradermal BoNT‐A microdroplets, 1 U per point at 1‐cm intervals, with a total dose of 50 U every 4 months, was initiated. Flushing and dysesthesia decreased within 4 weeks, and near‐complete resolution was observed at 10 months, accompanied by substantial improvement in quality of life. Weight gain of approximately 3 kg and mild somnolence occurred; after 6 months of treatment, paroxetine was reduced from 25 mg/day to 10 mg/day, and partial relapse of flushing and dysesthesia was observed approximately 4 weeks after dose reduction, requiring re‐escalation to 25 mg/day (Figure 1).

**FIGURE 1:**
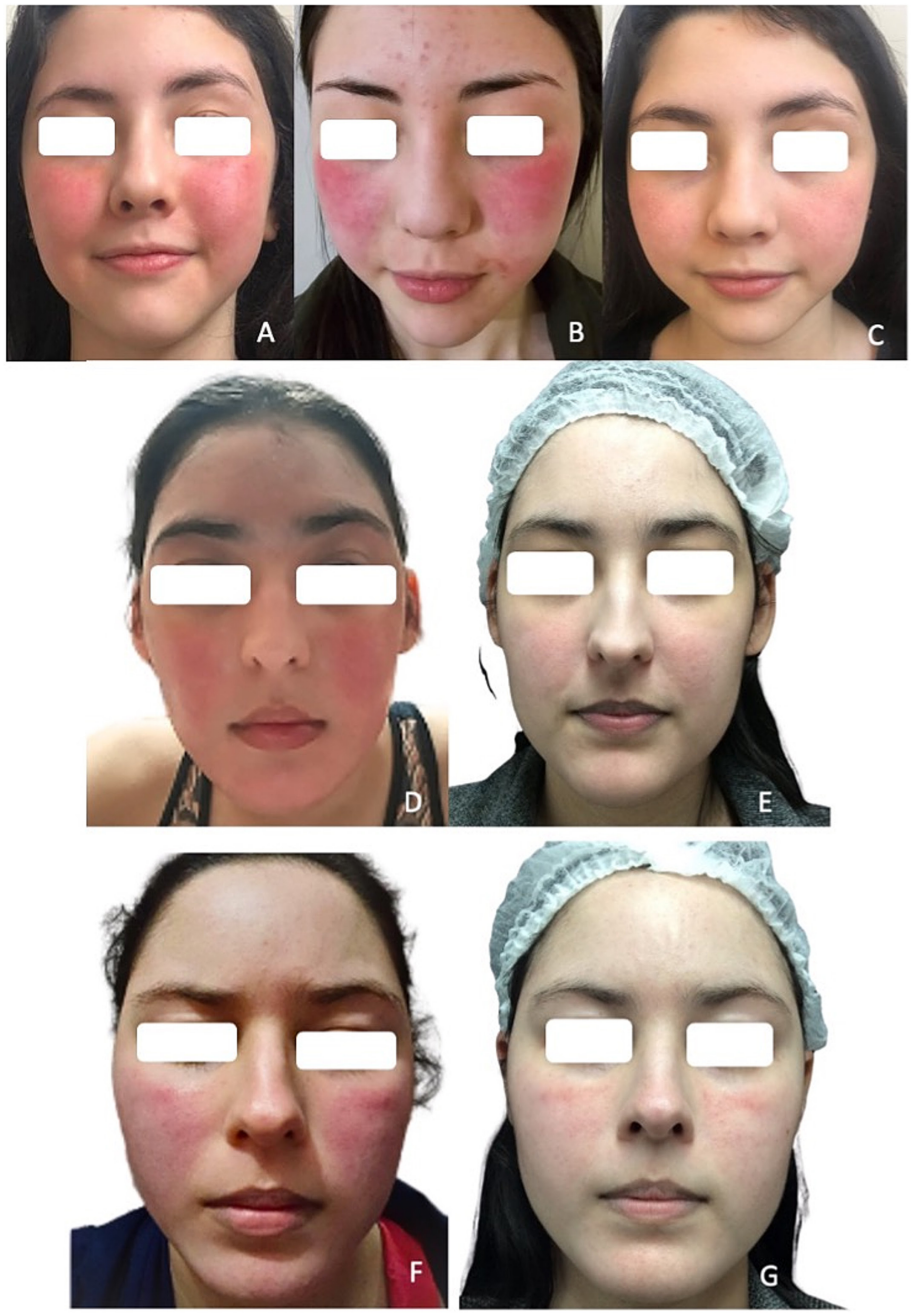
Clinical response of refractory erythematous rosacea to combined oral paroxetine and intradermal botulinum toxin type A. (A: Case 1; D and F: Case 2 twins) Baseline persistent facial erythema. (B) Worsening with previous treatments. (C, E and G) Marked reduction of erythema after combined treatment with paroxetine and intradermal botulinum toxin A.

These preliminary observations suggest that simultaneously targeting central and peripheral neurovascular pathways may enhance control of refractory erythematous rosacea. Paroxetine may modulate central vasomotor reactivity and anxiety‐related triggers, while BoNT‐A appears to improve rosacea through multimodal mechanisms beyond neuromodulation, including effects on vasodilation, neurogenic inflammation, and mast cell activity. This dual strategy may interrupt the self‐perpetuating loop of vascular instability, neurogenic inflammation, and emotional stress underlying chronic facial erythema. Controlled prospective studies are warranted to validate this approach and optimize treatment protocols.

## Funding

The authors have nothing to report.

## Ethics Statement

The patients in this manuscript have given written informed consent to publication of their case details.

## Conflicts of Interest

The authors declare no conflicts of interest.

## Data Availability

Data sharing not applicable to this article as no datasets were generated or analysed during the current study.
